# Ticagrelor Is Superior to Clopidogrel in Inhibiting Platelet Reactivity in Patients With Minor Stroke or TIA

**DOI:** 10.3389/fneur.2020.00534

**Published:** 2020-06-10

**Authors:** Yingying Yang, Weiqi Chen, Yuesong Pan, Hongyi Yan, Xia Meng, Liping Liu, Yongjun Wang, Yilong Wang

**Affiliations:** ^1^Department of Neurology, China National Clinical Research Center for Neurological Diseases, Beijing Tiantan Hospital, Capital Medical University, Beijing, China; ^2^Advanced Innovation Center for Human Brain Protection, Capital Medical University, Beijing, China

**Keywords:** clopidogrel, platelet reactivity, stroke, ticagrelor, transient ischemic attack

## Abstract

**Background:** The combination of clopidogrel and aspirin is recommended for the treatment of patients with acute minor stroke or transient ischemic attack (TIA). However, with varied clopidogrel resistance (often due to *CYP2C19* loss-of-function (LOF) alleles), alternatives like ticagrelor have been suggested. Previous studies showed that ticagrelor had a lower platelet reactivity assessed by VerifyNow P2Y12 assay than clopidogrel. We aimed to compare the effect of ticagrelor vs. clopidogrel on platelet reactivity assessed by a different method (Aggrestar platelet function analyzer) and analyze whether *CYP2C19* genotypes were involved.

**Methods:** A pre-specified subgroup analysis of a randomized controlled trial- Platelet Reactivity in Acute Non-disabling Cerebrovascular Events (PRINCE) was conducted. Patients with minor stroke or TIA were randomized for treatment with ticagrelor plus aspirin or clopidogrel plus aspirin. Platelet reactivity was assessed by Aggrestar (PL) platelet function analyzer and high on-treatment platelet reactivity (HOPR) on ticagrelor or clopidogrel was compared. Clinical outcomes included any stroke, composite vascular events and bleeding events within 90 days. Patients were categorized into carriers and non-carriers according to the carrier status of *CYP2C19* LOF alleles.

**Results:** Among 675 patients enrolled in the PRINCE trial, 387 patients were included in this subgroup: 197 were randomized to ticagrelor plus aspirin and 190 to clopidogrel plus aspirin. At 90 ± 7 days, compared with clopidogrel/aspirin group, the proportion of HOPR in ticagrelor/aspirin group was significantly lower (19.6 vs. 40.8%, *P* < 0.001). No significant treatment-by-genotype interactions were found (*P* for interaction = 0.12). Within 90 days, a trend toward a lower risk of new stroke in ticagrelor/aspirin compared to clopidogrel/aspirin was observed (4.6 vs. 9.5%, HR 0.47, 95% CI 0.21-1.05, *P* = 0.06).

**Conclusions:** Ticagrelor is superior to clopidogrel in inhibiting platelet reactivity measured by the PL platelet function analyzer among patients with acute minor stroke or TIA. Our study confirmed the finding of the main analysis of PRINCE trial in a different assay. Large randomized controlled trials are needed to evaluate our findings.

**Clinical Trial Registration:** Clinicaltrials.gov NCT02506140.

## Introduction

Acute minor ischemic stroke and high risk transient ischemic attack (TIA) are very common. The combination of clopidogrel and aspirin is recommended for secondary prevention of patients with acute minor stroke or TIA (1–5). However, the incidence of clopidogrel resistance ranges from 5 to 30% (6). Among many factors related to clopidogrel resistance, the *CYP2C19* loss-of-function (LOF) alleles, particularly, ^*^2 and ^*^3, are associated with poor clopidogrel metabolism (7, 8).

Ticagrelor, a reversible P2Y12 receptor antagonist, inhibits platelet reactivity irrespective of *CYP2C19* genotypes (9, 10). Previous studies demonstrated that ticagrelor is more reliable than clopidogrel in inhibiting platelet reactivity and reducing the recurrence of ischemic vascular events in patients with acute coronary syndrome (11–13). In recent years, more and more clinical trials have focused on the application of ticagrelor among patients with cerebrovascular events (14–16). However, whether ticagrelor is superior to clopidogrel in reducing stroke recurrence in patients with acute minor stroke or high-risk TIA remains unclear.

Platelet function tests can assess the response variability to antiplatelet therapies. High on-treatment platelet reactivity (HOPR) is thought to be strongly associated with ischemic events, and the proportion of HOPR could reflect the efficacy of antiplatelet therapy (17, 18). Platelet Reactivity in Acute Non-disabling Cerebrovascular Events (PRINCE) trial showed that ticagrelor had a lower proportion of HOPR assessed by VerifyNow P2Y12 assay than clopidogrel in patients with minor stroke and TIA (16). Aggrestar (PL) platelet function analyzer is a novel analyzer via sequential platelet counting method and it's cheap, quick and easy to monitor platelet function (19). In this pre-specified subgroup analysis of PRINCE trial, we aimed to investigate the comparative effects of ticagrelor plus aspirin vs. clopidogrel plus aspirin on platelet reactivity, which was assessed by the PL platelet function analyzer, and whether *CYP2C19* genotypes were involved in the process.

## Materials and Methods

### Study Population

A pre-specified subgroup analysis of PRINCE trial was conducted, which was a randomized, prospective, multicenter, open-label, active-controlled, blind-endpoint trial. The rationale and design of PRINCE trial have been described previously (20). Briefly, the trial randomized patients with acute minor ischemic stroke (National Institutes of Health Stroke Scale score ≤3), or moderate to high risk TIA (ABCD^2^ stroke risk score of ≥ 4 or ≥ 50% stenosis of cervical or intracranial vessels that could account for the presentation) to receive ticagrelor plus aspirin or clopidogrel plus aspirin within 24 h of symptoms onset. Participants received ticagrelor (180 mg on day 1, followed by 90 mg twice daily on days 2–90) or clopidogrel (300 mg on day 1, followed by 75 mg daily on days 2–90) with an aspirin background using (300 mg on day 1, followed by 100 mg daily on days 2–21). Patient enrolment began in China in August 2015, and patient follow-up was completed by June 2017. Of the 26 centers included in PRINCE trial, participants in 17 centers voluntarily participated in the subgroup in which the platelet reactivity was measured by the PL platelet analyzer. The trial was registered with ClinicalTrials.gov (NCT02506140).

### Measurement of Platelet Reactivity

Platelet reactivity was assessed using the PL platelet function analyzer (Sinnowa Medical Science & Technology Co., Nanjing, China) at baseline, 7 + 2 days and 90 ± 7 days after randomization. Arachidonic acid (AA) (2 mg/mL) or adenosine diphosphate (ADP) (50 μmol/L) were used as inductive agents, and 40 μL of each were added to the samples, which were then mixed to initiate platelet aggregation. The analyzer counted the platelets several times until the lowest level was detected. The maximum aggregation ratio (MAR) value was calculated following exposure to each inductive agent. HOPR on ticagrelor or clopidogrel was defined as MAR_ADP_ ≥ 35%, and HOPR on aspirin was defined as MAR_AA_ ≥ 35% (21). Considering that aspirin was only given for the first 21 days, we didn't evaluate MAR_AA_ at 90 ± 7 days. In addition, VerifyNow testing was conducted for these patients and P2Y12 reaction unit was measured at 90 ± 7 days (20).

At 7 + 2 days and 90 ± 7 days, blood for platelet function evaluation was sampled between 2 and 4 h after the morning maintenance dose of antiplatelet drugs. Platelet function needed to be assessed within 2 h after blood was sampled. Platelet function testing was conducted according to a standardized procedure manual in each study center by qualified personnel who were blinded to treatment allocation. Both the investigators and patients were aware of the study drug assignment, but were blinded to platelet reactivity data until the end of the trial. To ensure the validity and reproducibility of these methods, we held two separate training courses for all the testing personnel from each center.

### Clinical Outcomes

Efficacy outcomes included any stroke (ischemic/hemorrhagic) and composite vascular events (ischemic/hemorrhagic stroke, TIA, myocardial infarction, or vascular death) at 90 days. Safety outcomes included bleeding events at 90 days which were defined according to PLATO criteria (22).

### *CYP2C19* Genotyping

Blood samples were collected and shipped via cold-chain transportation from each center to Beijing Tiantan Hospital and stored at −80°C. Three *CYP2C19* single-nucleotide polymorphisms (SNPs) were assessed: CYP2C19^*^2 (681G > A, dbSNP rs4244285), CYP2C19^*^3 (636G > A, dbSNP rs4986893), and CYP2C19^*^17 (−806C > T, dbSNP rs12248560). Genotyping was performed on the Sequenom MassARRAY iPLEX platform (Sequenom, San Diego, California, USA) and Sanger sequencing (ABI 3500 Genetic Analyzer, Applied Biosystems, Foster City, CA, USA) if the results were otherwise inconclusive. The call rate of each SNP was >98.5%. Carriers of LOF alleles were defined as patients with at least one LOF allele (^*^2 or ^*^3), including the genotypes ^*^1/^*^2, ^*^1/^*^3, ^*^2/^*^2, ^*^2/^*^3, ^*^3/^*^3, ^*^2/^*^17, or ^*^3/^*^17. Non-carriers were defined as patients with no LOF alleles (^*^2 or ^*^3), including the genotypes ^*^1/^*^1, ^*^1/^*^17, or ^*^17/^*^17.

### Statistical Analysis

Continuous variables were presented as mean with standard deviation, or median with interquartile range, and categorical variables as percentages. Baseline characteristics were compared between ticagrelor/aspirin group and clopidogrel/aspirin group, using the Student's *t*-test or Wilcoxon test for continuous variables, and the χ^2^ test for categorical variables. We compared the proportion of HOPR between two treatment groups using generalized linear model, reported as a risk ratio (RR) with 95% confidence intervals (CI). At baseline, the proportion of HOPR was also compared after adjustment for age and sex. At 7 + 2 days and 90 ± 7 days, we also performed the analysis adjusted for age, sex, and HOPR status at baseline. We compared the correlation between the PL platelet analyzer and VerifyNow results by linear correlation. The differences in the rates of stroke, composite vascular events, ischemic stroke, and bleeding events during the 90 day follow-up were assessed by Cox proportional hazards regression, and were reported as hazard ratios (HR) with 95% CI. We also adjusted for age and sex. We assessed whether the treatment effect differed by testing the treatment-by-genotype interaction effect for the proportion of HOPR.

Two-sided *P* values < 0.05 were considered statistically significant. All analyses were performed using SAS 9.4 (SAS Institute, Cary, North Carolina).

## Results

### Study Participants and Baseline Characteristics

Among 675 patients enrolled in the PRINCE trial, 387 patients were included in this subgroup. Baseline characteristics of patients included and excluded in the subgroup are shown in Table 1. The median age of participants included in the subgroup was 61 years, and 28.4% of them were women. The index event was a minor stroke in 324 patients (83.7%) and a TIA in 63 patients (16.3%). Baseline characteristics were also compared between ticagrelor/aspirin group (197 patients) and clopidogrel/aspirin group (190 patients) and they were well balanced between two groups (Table 2).

**Table 1:** Baseline characteristics of participants included and excluded in our subgroup.

**Characteristics**	**Included (*N* = 387)**	**Excluded (*N* = 288)**	***P* value**
Age, y, median (IQR)	61 (55–67)	61 (54–67)	0.85
Female, *n* (%)	110 (28.4)	71 (24.7)	0.27
BMI, kg/m^2^, median (IQR)	24.5 (22.6–26.8)	24.9 (22.9–27.3)	0.19
Medical history, *n* (%)			
Hypertension	238 (61.5)	173 (60.1)	0.71
Dyslipidaemia	29 (7.5)	12 (4.2)	0.07
Diabetes mellitus	96 (24.8)	68 (23.6)	0.72
Ischemic stroke	73 (18.9)	48 (16.7)	0.46
TIA	11 (2.8)	7 (2.4)	0.74
Coronary artery disease	16 (4.1)	35 (12.2)	<0.0001
Current smoker	171 (44.2)	148 (51.4)	0.08
Drug use before randomization, *n* (%)			
Proton-pump inhibitor	4 (1.0)	1 (0.4)	0.30
Statin	49 (12.7)	17 (5.9)	0.004
Aspirin	97 (25.1)	49 (17.0)	0.01
Clopidogrel	10 (2.6)	5 (1.7)	0.46
Ticagrelor	0 (0.0)	0 (0.0)	-
Time from onset to randomization, h, median (IQR)	15.4 (8.5–20.8)	12.9 (7.7–20.6)	0.07
Qualifying event, *n* (%)			0.89
Minor stroke	324 (83.7)	240 (83.3)	
TIA	63 (16.3)	48 (16.7)	
NIHSS, median (IQR); mean ± SD	2(1–3); 1.60 ± 1.13	2(1–3); 1.63 ± 1.10	0.049
Baseline ABCD^2^ score, median (IQR)	5 (4,5)	5 (4,5)	0.91
SSS-TOAST stroke subtype, *n* (%)			0.44
Large-artery atherosclerosis	181 (55.9)	123 (51.3)	
Cardioaortic embolism	7 (2.2)	6 (2.5)	
Small-artery occlusion	113 (34.9)	100 (41.7)	
Other causes	11 (3.4)	5 (2.1)	
Undetermined causes	12 (3.7)	6 (2.5)	

*IQR, interquartile range; SD, standard deviation; BMI, body mass index; TIA, transient ischaemic attack; ABCD^2^, age, blood pressure, clinical features, duration of TIA, and presence of diabetes score squared; SSS-TOAST, Stop Stroke Study-Trial of Org 10172 in Acute Stroke Treatment*.

**Table 2:** Baseline characteristics of participants stratified by dual antiplatelet therapy.

**Characteristics**	**Ticagrelor/aspires (*N* = 197)**	**Clopidogrel/aspirin (*N* = 190)**	***P* value**
**NIHSS Median (IQR)**			
Age, y, median (IQR)	62 (55–67)	61 (55–67)	0.55
Female, *n* (%)	57 (28.9)	53 (27.9)	0.82
BMI, kg/m^2^, median (IQR)	24.3 (22.6–26.8)	24.8 (22.6–27.0)	0.50
Medical history, *n* (%)			
Hypertension	117 (59.4)	121 (63.7)	0.39
Dyslipidaemia	15 (7.6)	14 (7.4)	0.93
Diabetes mellitus	47 (23.9)	49 (25.8)	0.66
Ischaemic stroke	35 (17.8)	38 (20.0)	0.57
TIA	4 (2.0)	7 (3.7)	0.33
Coronary artery disease	10 (5.1)	6 (3.2)	0.34
Current smoker, *n* (%)	86 (43.7)	85(44.7)	0.91
Drug use before randomization, *n* (%)			
Proton-pump inhibitor	2 (1.0)	2 (1.1)	0.97
Statin	29 (14.7)	20 (10.5)	0.21
Aspirin	57 (28.9)	40 (21.0)	0.07
Clopidogrel	3 (1.5)	7 (3.7)	0.18
Ticagrelor	0 (0.0)	0 (0.0)	
Time from onset to randomization, h, median (IQR)	15.8 (8.7–20.8)	14.7 (8.3–20.7)	0.46
Qualifying event, *n* (%)			
Minor stroke	166 (84.3)	158 (83.2)	0.77
TIA	31 (15.7)	32 (16.8)	
NIHSS, median (IQR); mean ± SD	2(1–3); 1.62 ± 1.14	2(1–3); 1.58 ± 1.13	0.17
Baseline ABCD^2^ score, median (IQR)	5(4,5)	4.5 (4,5)	0.94
SSS-TOAST stroke subtype, *n* (%)			0.23
Large-artery atherosclerosis	100 (60.2)	81 (51.3)	
Cardioaortic embolism	5 (3.0)	2 (1.3)	
Small-artery occlusion	53 (31.9)	60 (38.0)	
Other causes	4 (2.4)	7 (4.4)	
Undetermined causes	4 (2.4)	8 (5.1)	

*IQR indicates interquartile range; SD, standard deviation; BMI, body mass index; TIA, transient ischaemic attack; ABCD^2^, age, blood pressure, clinical features, duration of TIA, and presence of diabetes score squared; SSS-TOAST, Stop Stroke Study-Trial of Org 10172 in Acute Stroke Treatment*.

### Effect of Ticagrelor/Aspirin vs. Clopidogrel/Aspirin on Platelet Reactivity

**Figure 1:**
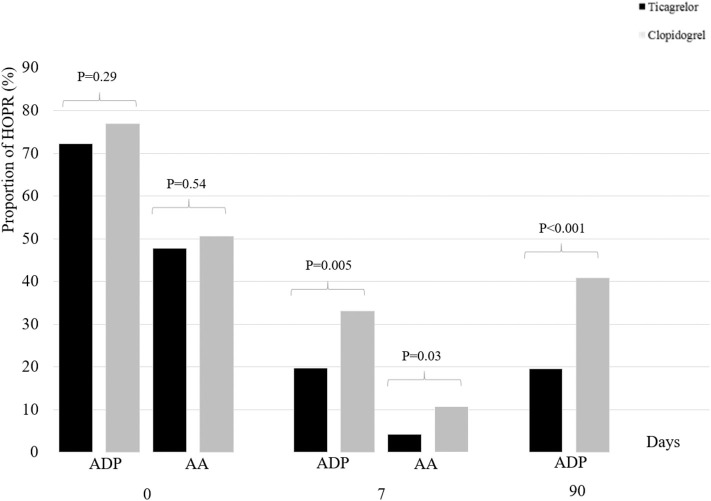
Proportion of HOPR stratified by dual antiplatelet therapy at baseline, 7 + 2 days and 90 ± 7 days. HOPR indicates high on-treatment platelet reactivity; AA, arachidonic acid; ADP, adenosine diphosphate.

At baseline, the proportion of HOPR between ticagrelor/aspirin group and clopidogrel/aspirin group was similar (MAR_ADP_, 72.2 vs. 76.9%, *P* = 0.29; MAR_AA_, 47.4 vs. 50.5%; *P* = 0.54). At 7 + 2 days, compared with clopidogrel/aspirin, the proportion of HOPR in ticagrelor/aspirin was significantly lower (MAR_ADP_, 19.8 vs. 33.0%; *P* = 0.005; MAR_AA_, 4.3 vs. 10.7%, *P* =0.03). At 90 ± 7 days, similar results were obtained as at 7 + 2 days (MAR_ADP_, 19.6 vs. 40.8%, *P* < 0.001) (Table 3, Figure 1). After adjustment for age, sex and HOPR status at baseline, similar results were obtained.

**Table 3:** Effect of ticagrelor/aspirin vs. clopidogrel/aspirin on platelet reactivity.

**HOPR**		**Ticagrelor/aspirin *N* (%) (*N* = 197)**	**Clopidogrel/aspirin *N* (%) (*N* = 190)**	**RR (95% CI)**	***P* value**	**Adjusted RR (95% CI)**	***P* value**
Baseline	MAR_ADP_ ≥35%	140/194 (72.2)	143/186 (76.9)	0.94 (0.83–1.06)	0.29	0.95(0.84–1.06)^#^	0.37
	MAR_AA_ ≥35%	90/190 (47.4)	94/186 (50.5)	0.94 (0.76–1.15)	0.54	0.93(0.76–1.15)^#^	0.53
7+2 days	MAR_ADP_ ≥35%	37/187 (19.8)	59/179 (33.0)	0.60 (0.42–0.85)	0.005	0.64(0.45–0.90)^*^	0.01
	MAR_AA_ ≥35%	8/186 (4.3)	19/177 (10.7)	0.40(0.17–0.86)	0.03	0.40(0.17–0.85)^*^	0.02
90 ± 7 days	MAR_ADP_ ≥35%	33/168 (19.6)	64/157 (40.8)	0.48 (0.33–0.68)	<0.001	0.48(0.33–0.68)^*^	<0.001

*HOPR indicates high on-treatment platelet reactivity; MAR, maximum aggregation ratio; AA, arachidonic acid; ADP, adenosine diphosphate; RR, risk ratio; CI, confidence interval*.

# *Adjusted for age and sex*.

* *Adjusted for age, sex and HOPR status at baseline*.

At 90 ± 7 days, there were 325 patients who evaluated platelet reactivity by both the PL platelet function analyzer and VerifyNow, and we found that MAR_ADP_ moderately correlated with P2Y12 reaction unit (Pearson correlation, *r* = 0.566, *P* < 0.001).

### Effect of Ticagrelor/Aspirin vs. Clopidogrel/Aspirin on Platelet Reactivity Stratified by *CYP2C19* LOF Allele Carrier Status

**Figure 2:**
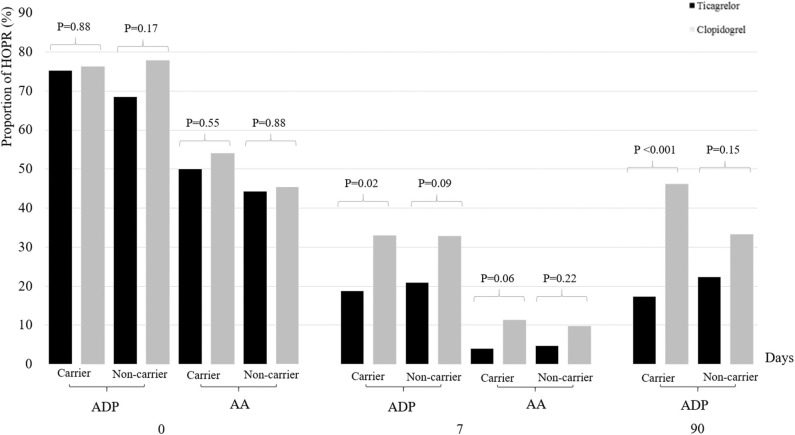
Proportion of HOPR stratified by dual antiplatelet therapy and *CYP2C19* loss-of-function allele carrier status at baseline, 7 + 2 days and 90 ± 7 days. HOPR indicates high on-treatment platelet reactivity; AA, arachidonic acid; ADP, adenosine diphosphate.

Among 387 patients with genetic data, 218 patients (56.3%) were *CYP2C19* LOF allele carriers and 169 (43.7%) were non-carriers. We did not identify any treatment-by-genotype interaction at the various time points (Table 4, Figure 2). At baseline, the proportion of HOPR was similar between two treatment groups, irrespective of the carrier status of *CYP2C19* LOF alleles. At 7 + 2 days, the proportion of HOPR on aspirin was not significantly different between two treatment groups among carriers (4.0 vs. 11.4%; *P* = 0.06) and non-carriers (4.7 vs. 9.7%; *P* = 0.22). Compared with clopidogrel/aspirin, ticagrelor/aspirin group had lower proportion of MAR_ADP_ ≥35% among carriers (18.8 vs. 33.3%, *P* = 0.02), but no significant difference was found among non-carriers (20.9 vs. 32.9%, *P* = 0.09). At 90 ± 7 days, similar results were obtained as at 7 + 2 days (carriers, 17.4 vs. 46.2%, *P* < 0.001; non-carriers, 22.4 vs. 33.3%, *P* = 0.15). However, no significant treatment-by-genotype interactions were found (*P* for interaction = 0.12). After adjustment for age, sex, and HOPR status at baseline, similar results were obtained.

**Table 4:** Effect of ticagrelor/aspirin vs. clopidogrel/aspirin on platelet reactivity stratified by *CYP2C19* loss-of-function allele carrier status.

**HOPR**					**Carriers**						**Non-carriers**			***P* for Interaction**
	**Ticagrelor/aspirin** ***N*** **(%) (*****N*** **=** **107)**		**Clopidogrel/aspirin *N* (%) (*N* = 111)**	**RR (95% CI)**	***P* value**	**Adjusted RR (95% CI)**	***P* value**	**Ticagrelor/ aspirin *N* (%) (*N* = 90)**	**Clopidogrel/Aspirin *N* (%) (*N* = 79)**	**RR (95% CI)**	***P* value**	**Adjusted RR (95% CI)**	***P* value**	
Baseline	MAR_ADP_ ≥35%	79/105 (75.2)	83/109 (76.2)	0.99(0.85–1.15)	0.88	1.01(1.00–1.16)^#^	0.93	61/89 (68.5)	60/77 (77.9)	0.88(0.73–1.06)	0.17	0.88(0.73–1.06)^#^	0.19	0.34
	MAR_AA_ ≥35%	51/102 (50.0)	59/109 (54.1)	0.92(0.71–1.20)	0.55	0.94(0.72–1.21)^#^	0.63	39/88 (44.3)	35/77 (45.5)	0.98(0.69–1.38)	0.88	0.99(0.70–1.41)^#^	0.97	0.8
7 + 2 days	MAR_ADP_ ≥35%	19/101 (18.8)	35/106 (33.0)	0.57(0.34–0.91)	0.02	0.61(0.37–0.96)*	0.04	18/86 (20.9)	24/73 (32.9)	0.64(0.37–1.07)	0.09	0.73(0.42–1.23)*	0.24	0.76
	MAR_AA_ ≥35%	4/100 (4.0)	12/105 (11.4)	0.35(0.10–0.97)	0.06	0.38(0.11–1.06)*	0.09	4/86 (4.7)	7/72 (9.7)	0.48(0.13–1.52)	0.22	0.48(0.13–1.51)*	0.22	0.7
90 ± 7 days	MAR_ADP_ ≥35%	16/92 (17.4)	42/91 (46.2)	0.38(0.22–0.60)	<0.001	0.38(0.22–0.61)*	<0.001	17/76 (22.4)	22/66 (33.3)	0.67(0.38–1.15)	0.15	0.70(0.39–1.22)*	0.22	0.12

*HOPR, high on-treatment platelet reactivity; MAR, maximum aggregation ratio; AA, arachidonic acid; ADP, adenosine diphosphate*.

Carriers, defined as patients who carried at least one CYP2C19 loss-of-function allele (*2 or *3).

Non-carriers, defined as patients who did not carry either of CYP2C19 loss-of-function alleles (*2 or *3).

*RR, risk ratio; CI, confidence interval*.

# *Adjusted for age and sex*.

* *Adjusted for age, sex and HOPR status at baseline*.

### Effect of Ticagrelor/Aspirin vs. Clopidogrel/Aspirin on Clinical Outcomes

At 90 ± 7 days, a trend toward a lower risk of new stroke in ticagrelor/aspirin compared to clopidogrel/aspirin was observed (4.6 vs. 9.5%, HR 0.47, 95% CI 0.21-1.05, *P* = 0.06). Compared with clopidogrel/aspirin, ticagrelor/aspirin reduced the occurrence of composite events (5.1 vs. 10.5%, HR 0.47, 95% CI 0.22-1.00, *P* = 0.050) and ischemic stroke (3.6 vs. 9.0%, HR 0.39, 95% CI 0.16-0.93, *P* = 0.03). No significant differences in the risk of major bleeding or any bleeding were observed between two groups (Table 5). After adjustment for age and sex, similar results were obtained.

**Table 5:** Effect of ticagrelor/aspirin vs. clopidogrel/aspirin on clinical outcomes within 90 days.

**Outcome**	**Ticagrelor/Aspirin N (%) (*N* = 197)**	**Clopidogrel/Aspirin *N* (%) (*N* = 190)**	**HR (95%CI)**	***P* value**	**Adjusted HR (95%CI)^#^**	***P* value**
**Efficacy outcome**						
Stroke	9(4.6)	18(9.5)	0.47(0.21–1.05)	0.06	0.47(0.21–1.04)	0.06
Composite vascular events	10(5.1)	20(10.5)	0.47(0.22–1.00)	0.050	0.47(0.22–1.00)	0.049
Ischemic stroke	7(3.6)	17(9.0)	0.39(0.16–0.93)	0.03	0.38(0.16–0.92)	0.03
**Safety outcome**						
Major bleeding	2(1.0)	2(1.1)	0.97(0.14–6.87)	0.97	1.02(0.14–7.29)	0.98
Any Bleeding	39(19.8)	25(13.2)	1.57(0.95–2.59)	0.08	1.57(0.95–2.60)	0.08

*CI indicates confidence interval*.

# *Adjusted for age and sex*.

## Discussion

The pre-specified subgroup analysis of the PRINCE trial confirmed the finding of the main analysis with the PL platelet function analyzer: ticagrelor is superior to clopidogrel in inhibiting platelet reactivity among patients with acute minor stroke or TIA. Our study also afforded more evidence for the application of the PL platelet function analyzer in assessing the efficacy of antiplatelet therapies in these patients.

The PL subgroup analysis has made a consistent conclusion with the main analysis of the PRINCE trial in which 675 patients were enrolled and platelet reactivity was assessed by VerifyNow P2Y12 assay (16). The VerifyNow P2Y12 assay is the most widely described method in clinical trials to assess platelet reactivity to clopidogrel or ticagrelor (17, 23, 24). Our study also showed that PL platelet function analyzer results moderately correlated with VerifyNow, suggesting that the new method is reliable in assessing the treatment effects of antiplatelet drugs. Among patients undergoing percutaneous coronary intervention, HOPR measured by the PL platelet function analyzer was associated with incremental stent thrombosis (25). The cut-off values (MAR_ADP_ ≥ 35%, MAR_AA_ ≥ 35%) had been previously confirmed to predict a higher risk of ischemic stroke or TIA (21). In addition, considering that the method is cheap, quick and easy to monitor platelet function, PL platelet function analyzer might be widely used in clinical practice.

The conversion of clopidogrel to its active metabolite is regulated by the cytochrome P450 system, especially *CYP2C19*. The findings from a meta-analysis showed that carriers of *CYP2C19* LOF alleles had attenuated responses to clopidogrel in patients with acute minor stroke or TIA (8). Ticagrelor does not require metabolic activation after ingestion, and inhibits platelet reactivity directly. Our study found that ticagrelor had a lower platelet reactivity than clopidogrel among carriers, but not among non-carriers. We speculated that the superiority of ticagrelor over clopidogrel in inhibiting platelet reactivity among carriers of *CYP2C19* LOF alleles was mainly associated with reduced clopidogrel metabolism. Carriers of *CYP2C19* LOF alleles might achieve more benefit with ticagrelor than clopidogrel. It might be necessary to conduct genetic testing before an individualized antiplatelet therapy was given (12). However, our results showed that there was no significant interaction between treatment groups and the carrier status of *CYP2C19* LOF alleles, and this may be related to limited sample size. Large randomized controlled trials are needed to evaluate this.

Our study also found that ticagrelor plus aspirin reduced the proportion of HOPR on aspirin compared with clopidogrel plus aspirin at 7 + 2 days. The platelet aggregation induced by AA depends on the activity of the cyclo-oxygenase-1 (COX-1) enzyme, and aspirin irreversibly inhibits this enzyme. Both ticagrelor and clopidogrel inhibit the P2Y12 receptor, which is the downstream of the COX-1 pathway. Our results indicated that ticagrelor might be superior to clopidogrel not only in inhibiting the platelet aggregation induced by ADP, but also in inhibiting the platelet aggregation induced by AA.

There were several advantages in our study. First, the data was from a randomized controlled trial, thus diminishing the effect of potential confounding factors as much as possible. Second, both platelet reactivity and clinical outcomes were analyzed to investigate the impact of ticagrelor vs. clopidogrel on platelet function among patients with minor stroke or TIA. There were several limitations. First, some factors might affect the interpretation of platelet reactivity assessed by PL platelet function analyzer, such as the coagulation function, mean platelet volume, and the number of platelets before the measurements. Second, about 15% of the patients were lost to follow-up for the evaluation of platelet function at 90 days. However, similar results were observed after assuming that all the missing data were HOPR, or not. Third, baseline characteristics of patients included and excluded in this subgroup were not totally balanced, including the proportions of patients with coronary artery disease and pre-randomization statin and aspirin use, and NIHSS score. Therefore, the generalization of our results is limited and more randomized trails are needed to confirm them.

In summary, ticagrelor is superior to clopidogrel in inhibiting platelet reactivity measured by the PL platelet function analyzer among patients with acute minor stroke or TIA. Our study confirmed the finding of the main analysis of PRINCE trial in a different assay. Large randomized controlled trials are needed to evaluate our findings.

## Data Availability Statement

The datasets generated for this study can be directed to Prof Yilong Wang, yilong528@gmail.com.

## Ethics Statement

The study protocol and data collection were approved by the ethics committee of Beijing Tiantan Hospital (ethical approval number KY2014-048-03) and all of the study centers, and conducted in accordance with the Declaration of Helsinki. All of the patients provided their written informed consent to participate in this study.

## Author Contributions

YY and WC participated in study design and drafted the manuscript. XM, LL, and YoW participated in acquisition of data and the design of the study. YP and HY participated the statistical analysis. YiW participated in the design of the study and helped to draft the manuscript. All authors have read and approved the submitted manuscript.

## Conflict of Interest

The authors declare that the research was conducted in the absence of any commercial or financial relationships that could be construed as a potential conflict of interest.
